# Medical Assistance in Dying, Data and Casting Assertions Aside

**DOI:** 10.1089/jpm.2022.0484

**Published:** 2022-12-30

**Authors:** Harvey Max Chochinov

**Affiliations:** ^1^ Distinguished Professor of Psychiatry, University of Manitoba, Winnipeg, Manitoba, Canada.; ^2^ Senior Scientist, CancerCare Manitoba Research Institute, Winnipeg, Manitoba, Canada.

**Keywords:** assertions, data, ethics, MAiD, research, transparency

## Abstract

Various assertions have been made regarding why eligibility for medical assistance in dying (MAiD) should be expanded. Examining these and the studies used to support them should clear the way for thoughtful data monitoring and research into why some patients make death hastening requests. This will not only improve MAiD practices in Canada, but will lead to better more effective palliative care for patients whose suffering leads them to covet death.

In 2015, I was appointed Chair of the Canadian Federal Government's External Panel for a Legislative Response to Carter versus Canada.^1^ Our panel was tasked with reviewing all facets of euthanasia and assisted suicide, to help inform and guide parliamentarians in crafting new legislation. Our mandate included an environmental scan, gleaning lessons from constituencies around the world already permitting some form of physician hastened death. Six years later, the eyes of the world are now on Canada; eyes that no doubt see how policies originally designed to assist dying patients hasten their death have morphed into legislation that is no longer restricted to those nearing death, providing access to patients with incurable, although not imminently fatal illness, disease or disability, and starting in March 2023, those whose sole underlying medical condition is mental illness.

Some interesting arguments and rational for expanding Medical Assistance in Dying (MAiD) in Canada were presented at a recent two-day symposium I attended entitled, *Medical Assistance in Dying (MAiD) in Canada: A Multidisciplinary Pan-Canadian Knowledge Translation Initiative to Improve Practice Documents and Plan a Research Agenda on Suffering in the Context of a MAiD Request*. This McGill University hosted pan-Canadian meeting, sponsored by the Canadian Institutes of Health Research and The Réseau québécois de recherche en soins palliatifs et de fin de vie, involved multiple clinicians and scientists in the field of MAiD, palliative care, and suicide prevention.

Other attendees included delegates from Health Canada, the Canadian Institute of Health Information, different professional associations, and government representatives. Having listened very closely to the proceedings, I come away with a deeper understanding of some assertions being made by those promoting expanded access to MAiD, and how data are used to support their position.

The first assertion of MAiD proponents is that MAiD is not driven by an inability to access palliative care. Although reference is made to the 2020 Health Canada Report on MAiD,^2^ which indicates that 82.8% of MAiD recipients obtain palliative care services, there seems little concern that the only information collected about palliative care for MAiD recipients is whether it was received or not and for how long. These data, submitted by MAiD providers, are silent on what defines, and what minimal threshold constitutes “receiving palliative care.” Imagine a patient who, after two years of eschewing health care for fear of being exposed to COVID-19, presents with widely metastatic cancer, associated with profound pain and suffering due to feeling a burden to his family, having received no supports whatsoever. A palliative care consultation recommends aggressive analgesia and supports for total pain, despite which he insists on having MAiD. Would such a patient be deemed a palliative care recipient, or more accurately, categorized as the epitome of failure to provide early and consistent palliation? Suffering, similar to cancer, grows, spreads, and if left unchecked, can kill. The Health Canada report,^2^ indicating 37.1% of MAiD patients receive palliative care for less than one month, and 54.8% greater than one month (with 8.1% unknown) offers insufficient data to affirm that these patients are being palliated in a manner designed to keep their suffering in check.

Another study intended to reinforce that MAiD has nothing to do with access to palliative care, reports the early experience of MAiD in Ontario, indicating that “palliative care providers were involved at any point in the care of 77.2% of patients, and at the time of the request for MAiD in 74.4%.”^3^ Similar to the Health Canada report,^2^ this study reports the involvement of palliative care of any kind, without any specificity whatsoever of what that means. This is akin to reporting how many people took a drug, although neglecting to provide the dose, the route, and duration of administration. Another observation made in support of this assertion is that patients with cancer, compared with those with organ failure or frailty, are far more likely to receive any palliative care, community-based palliative care, and do so for much longer periods of time.^4^

Although this study says nothing about MAiD, it is used to argue that if the provision of MAiD was driven by limited access to palliative care or unmet palliative care needs, we simply would not see more than three times the rate of MAiD in cancer compared with noncancer illness. This fails to say that a predictable trajectory of advanced cancers means these patients are usually eligible for most palliative care services; and under Canada's original C-14 legislation—requiring a reasonably foreseeable death—meet eligibility criteria for MAiD. In contrast, clinicians often struggle or are reticent to prognosticate with confidence in nonmalignant progressive life-limiting conditions; whereas patients with chronic noncancer conditions often have life expectancies that exceed reasonably foreseeable death. In other words, the lower uptake of MAiD in noncancer patients has little to do with their palliative care utilization, but rather to the fact that they either are not, or are not identified as being, eligible.

A final argument disconnecting access to palliative care and MAiD observes that countries with the highest opioid utilization are those with the highest rates of MAiD. If availability of opioids, as a proxy for access to palliative care, averted the need for MAiD, proponents argue that the data ought to move in exactly the opposite direction. This fails to even scratch the surface of complexity regarding the international availability of opioids, the profound sociopolitical differences between developed and developing countries, and the global disparities in the organization, provision, and quality of health care.

Between 2011 and 2013, greater than 95% of global opioid analgesic use occurred in a small number of high-income countries, accounting for only 15% of the global population, whereas the use of opioid analgesics in other parts of the world is disproportionately low and does not meet the basic needs for pain control.^5^ Latin America consumes <1% of the world's morphine.^6^ Colombia is unique, in that despite limited access to opioids resulting from deficiencies in the procurement processes, insufficient human resources, excessive bureaucratic tasks, insufficient number of pharmacies authorized to dispense controlled medications, lack of training in health care professions, and overly restrictive laws and regulations governing opioid availability, assisted suicide and euthanasia were decriminalized for patients more than six years old.^6–8^

Colombia notwithstanding, countries with low access to opioids, for various complex geopolitical, cultural, economic, and historical reasons, have not legalized euthanasia or assisted suicide. In other words, the low uptake of death hastening options in developing countries has little to do with opioid availability, but rather, simply reflects these life-ending alternatives are not allowed.

The next assertion used to promote expanded access to MAiD is that requests are not primarily driven by physical suffering. This argument is meant to neutralize the potential influence palliative care might have in mitigating suffering that manifests as a wish to die. In other words, if palliative care can address physical suffering, and the latter is seen as a driver of requests for hastened death, MAiD would seem a less attractive option. Health Canada reports 57.6% of MAiD recipients have inadequate pain control (or concern), and 46% inadequate control of symptoms other than pain (or concern).^4^ A study of Ontarians receiving MAiD reported 99.5% had physical suffering.^2^ Along with minimizing the potential influence of physical suffering on MAiD, the case is made that palliative care has limited ability to offer relief.

For example, a study of family proxies is highlighted, reporting 47.5% of their decedents who received specialized palliative care services (SPCS) in the last two weeks of life were somewhat (20.4%) or very (27.1%) uncomfortable.^9^ The study authors point out that people with more complex symptoms are more likely to be referred to SPCS, and that families tend to rate symptoms more severely than patients. Another study used to curb the promise of palliative care to deliver comfort illustrates high symptom burden toward death.^10^ However, many of the prevalent symptoms reported in the study during the final seven days of life, such as loss of appetite, drowsiness, fatigue, sleep, urinary/fecal incontinence, dysphagia to solids and liquids, may represent not so much the limitations of palliative care, but the inevitable consequences of what happens to bodies as they are dying.^10^

It is also asserted that existential suffering is the most profound influence on requests for MAiD. Health Canada data indicate that *loss of ability to engage in meaningful activities* (86.3%) and *loss of ability to perform activities of daily living (83.4%)* are the most prominent sources of suffering for those receiving MAiD.^4^ Although proponents characterize these as existential suffering, loss of the ability to engage in meaningful activities or activities of daily living implicates various and diverse challenges, including symptom distress, functional decline, and grief associated with diminished autonomy.

The Health Canada data delineating sources of suffering for patients receiving MAiD indicate that 3% experience *emotional distress/anxiety/fear/existential suffering.*^2^ The typology of existential distress, such as fractured dignity, is complex, and requires a deep understanding of, and attentiveness to, physical, psychosocial, and spiritual assaults that can undermine personhood; this is fundamental to various approaches addressing existential suffering in patients nearing death.^11^

Having elevated the prominence of existential distress as a driver of MAiD, proponents assert that there is little to be done about this particular driver of a wish to die. One witness, supporting expanded MAiD eligibility, told the Special Joint Parliamentary Committee on MAiD that, “this is a type of suffering for which we have very little or nothing to offer.”^12^ Decades of research, clinical practice, and academic inquiry appear to have no standing in making this assertion.^13^ Instead, the evidence used to support this argument includes a meta-analysis of randomized controlled trials of existential interventions on spiritual, psychological, and physical well-being in adult patients with cancer.^14^

Although some—trying to emphasize how little there is, aside from MAiD, to address existential suffering—have characterized the findings as unimpressive, the effect sizes for existential well-being (0.52), quality of life (0.21), hope (0.43), and self-efficacy (0.5) are not inconsiderable. The study authors conclude that their “analysis provides evidence that adult patients with cancer across all stages and types benefit from existential interventions,”^14^ whereas the editors of the special issue this study appeared in conclude that this analysis demonstrates “clear benefits arise from existentially oriented psychotherapy.”^15^

In the absence of targeted research and detailed data collection, assertions all too easily fill in the gap. Although there are widely differing opinions on MAiD, there are places we ought to find common ground.

1. The patient's voice must routinely be included in MAiD research and data monitoring.
2. We need a deeper understanding of what causes patients to want MAiD.
3. We need detailed data about psychosocial, supportive, palliative, existential, and spiritual measures that patients are offered and/or provided over the course of their condition leading to MAiD requests.
4. We must continue to develop and evaluate ways of addressing suffering that leads people to want to hasten death.

Health Canada states that, “federal public reporting on Medical Assistance in Dying continues to be a critical component to support transparency and foster public trust in the application of the law.”^16^ Although they acknowledge the need for the “consistent collection of information and public reporting,”^16^ how can there be transparency or public trust when no patient-level data whatsoever are being gathered? Currently, the only data collected by the Government of Canada for patients receiving MAiD is a form that takes nine minutes, on average according to Health Canada, for MAiD providers to complete.^17^ Surely understanding why patients want MAiD warrants more time than that. It is time to cast assertions aside; and instead, conduct research and gather data needed to fully understand requests for MAiD. This will not only improve MAiD practices in Canada, but will enhance our ability to provide better and more effective palliative care for patients whose suffering leads them to covet death.

## References

[1] Consultation on Physician-Assisted Dying: Summary of Results and Key Findings. Final Report. December 15, 2015. Available from: https://www.justice.gc.ca/eng/rp-pr/other-autre/pad-amm/pad.pdf [Last accessed October 12, 2022].

[2] Second Annual Report on Medical Assistance in Dying in Canada 2020. Available from: https://www.canada.ca/en/health-canada/services/medical-assistance-dying/annual-report-2020.html [Last accessed: August 21, 2020].

[3] Downar J, Fowler RA, Halko R, et al. Early experience with medical assistance in dying in Ontario, Canada: A cohort study. CMAJ 2020;192(8):E173–E181. 32051130 10.1503/cmaj.200016 PMC7043822

[4] Kendzerska T, Nickerson JW, Hsu AT, et al. End-of-life care in individuals with respiratory diseases: A population study comparing the dying experience between those with chronic obstructive pulmonary disease and lung cancer. Int J Chron Obstruct Pulmon Dis 2019;14:1691–1701. 31534323 10.2147/COPD.S210916 PMC6681558

[5] Berterame S, Erthal J, Thomas J, et al. Use of and barriers to access to opioid analgesics: A worldwide, regional, and national study. Lancet 2016;387:1644–1656. 26852264 10.1016/S0140-6736(16)00161-6

[6] Pain & Policy Studies Group. Availability of Opioid Analgesics in Latin America and the World. Guadalajara, Mexico; 2002. Prepared for: 1st Congress of the Latin American Association of Palliative Care, 7th Latin American Course on Medicine and Palliative Care; March 20–22.

[7] Leon MX, De Lima L, Florez S, et al. Improving availability of and access to opioids in Colombia: Description and preliminary results of an action plan for the country. J Pain Symptom Manage 2009;3:758–766. 10.1016/j.jpainsymman.2009.03.007 19783400

[8] World Federation Right to Die Societies. Available from: https://wfrtds.org/worldmap/colombia/ [Last accessed: July 31, 2022].

[9] Currow DC, Ward AM, Plummer JL, et al. Comfort in the last 2 weeks of life: Relationship to accessing palliative care services. Support Care Cancer 2008;16:1255–1263. 18335259 10.1007/s00520-008-0424-2

[10] Hui D, dos Santos R, Chisholm GB, et al. Symptom expression in the last seven days of life among cancer patients admitted to acute palliative care units. J Pain Symptom Manage 2015;50:488–494. 25242021 10.1016/j.jpainsymman.2014.09.003 PMC4366352

[11] Chochinov HM, Hassard T, McClement S, et al. The landscape of distress in the terminally ill. J Pain Symptom Manage 2009;38:641–649. 19713069 10.1016/j.jpainsymman.2009.04.021

[12] AMAD Committee Meeting, Parliament of Canada. Available from: https://parl.ca/DocumentViewer/en/44-1/AMAD/meeting-4/evidence#Int-11644568 [Last accessed: August 21, 2022].

[13] Chochinov HM, Breitbart W. (eds.) The Handbook of Psychiatry in Palliative Medicine. Oxford University Press; 2012.

[14] Bauereiß N, Obermaier S, Özünal SE, et al. Effects of existential interventions on spiritual, psychological, and physical well-being in adult patients with cancer: Systematic review and meta-analysis of randomized controlled trials. Psychooncology 2018;27:2531–2545. 29958339 10.1002/pon.4829

[15] Vehling S, Kissane DW. Existential distress in cancer: Alleviating suffering from fundamental loss and change. Psychooncology 2018;27:2525–2530. 30307088 10.1002/pon.4872

[16] Third Annual Report on Medical Assistance in Dying in Canada; 2021. Available from: https://www.canada.ca/en/health-canada/services/medical-assistance-dying/annual-report-2021.html [Last accessed August 21, 2020].

[17] Canada Gazette, Part I, Volume 156, Number 21: Regulations Amending the Regulations for the Monitoring of Medical Assistance in Dying. Available from: https://canadagazette.gc.ca/rp-pr/p1/2022/2022-05-21/html/reg1-eng.html [Last accessed: August 21, 2022].

